# Phorbol-12-myristate-13-acetate is a potent enhancer of B cells with a granzyme B^+^ regulatory phenotype

**DOI:** 10.3389/fimmu.2023.1194880

**Published:** 2023-07-27

**Authors:** Johanna Veh, Charlotte Mangold, Anja Felsen, Carolin Ludwig, Lisa Gerstner, Peter Reinhardt, Hubert Schrezenmeier, Dorit Fabricius, Bernd Jahrsdörfer

**Affiliations:** ^1^ Department of Transfusion Medicine, Ulm University, Ulm, Germany; ^2^ Institute for Clinical Transfusion Medicine and Immunogenetics, German Red Cross Blood Transfusion Service Baden-Württemberg–Hessen and University Hospital Ulm, Ulm, Germany; ^3^ Department of Pediatrics, University Medical Center Ulm, Ulm, Germany

**Keywords:** PMA, TPA, 12-O-tetradecanoylphorbol-13-acetate, regulatory B cells, GraB cells, granzyme B, GvHD

## Abstract

**Introduction:**

The infusion of *ex-vivo*-generated regulatory B cells may represent a promising novel therapeutic approach for a variety of autoimmune and hyperinflammatory conditions including graft-versus-host disease.

**Methods:**

Previously, we developed a protocol for the generation of a novel population of regulatory B cells, which are characterized by secretion of enzymatically active granzyme B (*GraB cells*). This protocol uses recombinant interleukin 21 (IL-21) and goat-derived F(ab)’2 fragments against the human B cell receptor (anti-BCR). Generally, the use of xenogeneic material for the manufacturing of advanced therapy medicinal products should be avoided to prevent adverse immune reactions as well as potential transmission of so far unknown diseases.

**Results:**

In the present work we demonstrated that phorbol-12-myristate-13-acetate (PMA/TPA), a phorbol ester with a particular analogy to the second messenger diacylglycerol (DAG), is a potent enhancer of IL-21-induced differentiation of pre-activated B cells into *GraB cells*. The percentage of *GraB cells* after stimulation of pre-activated B cells with IL-21 and PMA/TPA was not significantly lower compared to stimulation with IL-21 and anti-BCR.

**Discussion:**

Given that PMA/TPA has already undergone encouraging clinical testing in patients with certain haematological diseases, our results suggest that PMA/TPA may be a safe and feasible alternative for *ex-vivo* manufacturing of *GraB cells*.

## Introduction

**Figure 1:**
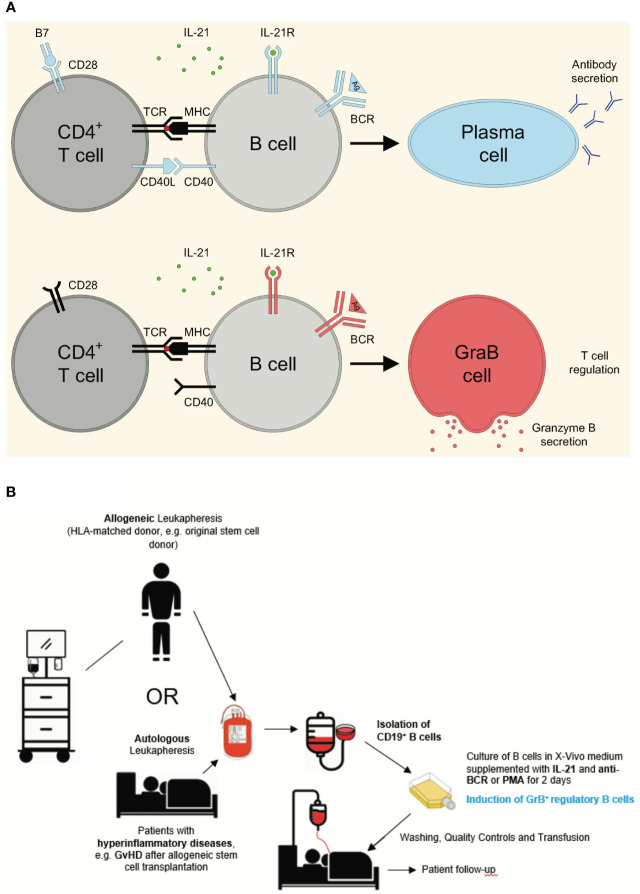
Differentiation of pre-activated B cells into either plasma cells or GrB^+^ regulatory *GraB cells* and concept of generation and application of GrB^+^ regulatory B cells for the treatment of hyper-inflammatory diseases. **(A)** Regular CD4^+^ T cell activation includes stimulation of both the T cell receptor (TCR) via MHC/peptide complexes and CD28 via B7. Such fully activated T cells secrete IL-21 and express high levels of CD40 ligand (CD40L), enabling them to induce plasma cell differentiation in B cells, when they receive antigen-specific signals via their B cell receptor (BCR) in parallel. In contrast, in certain pathological situations such as during an HIV infection, the TCR of CD4^+^ T cells can be directly stimulated (e.g. via the protein Nef during HIV infection), without simultaneous costimulation of CD28. Such incompletely activated T cells secrete IL-21, but barely express CD40L, resulting in the induction of GrB-secreting *GraB cells* instead of plasma cells. Experimentally, exogenous addition of CD40L multimers can substitute for incomplete B cell/T cell interactions, resulting in enhanced plasma cell differentiation *in vitro*. **(B)** GrB^+^ regulatory B cells (*GraB cells*) can be generated from either autologous or allogeneic, HLA-matched donor blood. After autologous or allogeneic leukapheresis B cells are enriched, for example by magnetic depletion of CD3^+^ T cells, followed by positive magnetic selection of CD19^+^ cells using MACS technology. Subsequently, B cells are cultured in a clean room environment (A-in-B clean room environment) in the presence of IL-21, the key cytokine for the generation of *GraB cells*. In order to achieve robust stimulation of alloreactive pre-activated B cells, a second stimulus is required, which can either directly target the B cell receptor or subordinate signaling pathways downstream of the B cell receptor as discussed later. After an incubation period of 48 hours, alloreactive pre-activated B cells express GrB and can be harvested from the incubation flasks. After several washing steps to remove remnants of stimulatory agents in the medium, cell suspensions are quality-tested to ensure purity, absence of endotoxin and microbiological contamination including mycoplasma. Then, the final cell product can be released and either cryopreserved or directly transfused to the patient. Potential indications are hyper-inflammatory diseases including autoimmune or graft-versus-host diseases. Anti-BCR, antibodies directed against the constant part of the B Cell Receptor; B7, Costimulatory molecules CD80 and CD86; BCR, B Cell Receptor; CD, Cluster of Differentiation; CD40L, CD40 Ligand; GvHD, Graft-versus-Host Disease; HLA, Human Leukocyte Antigen; IL-21, Interleukin 21; IL-21R, IL-21 Receptor; MACS, Magnetic Activated Cell Sorting; MHC, Major Histocompatibility Complex; PMA, Phorbol Myristate Acetate; TCR, T Cell Receptor.

As producers of highly specific antibodies, B cells constitute one of two central pillars of adaptive immunity against infectious agents and neoplastic cells. Nevertheless, via production of autoantibodies they may also be pathogenetically involved in autoimmune processes such as lupus erythematosus (SLE) (1) or GvHD (2). This is one of the reasons why counteractive B cell populations with anti-inflammatory and immunosuppressive potential have evolved in parallel. Such regulatory B cells exhibit various different phenotypes and exert a broad spectrum of effects in the context of hyperinflammatory, neoplastic and infectious diseases (3–10). In 2002, Fillatreau and colleagues described interleukin 10 (IL-10) as a central factor of one specific type of immunoregulatory B cells (11). IL-10^+^ B cells are referred to as B10 cells and have dominated the literature in the field of regulatory B cells since then. Between 2006 and 2015, our own laboratory discovered and described a novel population of regulatory B cells, which are characterized by production and secretion of the cytotoxic molecule granzyme B (GrB) (12–16). GrB^+^ regulatory B cells are referred to as *GraB cells*, are primarily IL-21-dependent and have the ability to negatively regulate T cells in a GrB-dependent manner (13–16). IL-21 is a pleiotropic cytokine (17), which can trigger B cell differentiation into either antibody-secreting plasma cells (18, 19), or into *GraB cells* (13–16). It is the integration of three signals acting on (1) the B cells´ IL-21 receptor, (2) on CD40, and (3) on the B cell receptor (BCR), which determines the final direction of B cell differentiation (**Figure 1A**). Previous work demonstrated that the combined action of IL-21 and antigen-specific stimulation of the BCR in the absence of CD40 ligation results in strong induction of GrB expression in B cells (12–16, 20).

In addition to its well-known cytotoxic functions, GrB can also mediate immunosuppressive effects. A prominent example is an acute infection with HIV, where NEF-dependent incomplete activation via the T cell receptor results in functional impairment of CD4^+^ T helper cells. As a consequence, CD4^+^-derived IL-21 in the absence of sufficient CD40 ligation promotes enhanced differentiation of B cells into *GraB cells*. The *GraB cells* in return foster a degradation of the TCR-ζ chain of CD4^+^ T cells, resulting in a vicious circle of rising numbers of *GraB cells* and a progressive loss of functional CD4^+^ T cells (16). The combined loss of functional T cells, antibody production and memory B cells eventually results in a severe depression of the affected individual’s immunological potential to properly respond to opportunistic infections, vaccination and neoantigens.

Numerical changes of GrB and IL-21 levels have also been found in the context of hyperinflammatory conditions such as in autoimmune and rheumatic diseases (21). In the case of SLE, not only elevated levels of IL-21 and GrB have been described, but also increased numbers of *GraB cells*, possibly reflecting a disease-modifying effect of these cells in SLE and other autoimmune diseases (21). Hyperinflammatory responses and their modulation by various immunoregulatory mechanisms are also relevant in the context of organ transplantation. Chesneau and colleagues found that the formation of long-term tolerance in renal transplant patients is associated with the presence of *GraB cells*. Tolerant recipients were shown to have reduced numbers of antibody-producing plasma cells. At the same time, they exhibited higher numbers of *GraB cells* compared to patients with chronic antibody-mediated graft rejection (22, 23). A similar picture is seen in the case of graft-versus-host disease (GvHD) after hematopoietic stem cell transplantation. Not only was the occurrence of chronic GvHD shown to be accompanied by aberrant IL-21 and IL-10 signaling and a decreased number of regulatory B cells. Also, the severity of chronic GvHD was negatively correlated with regulatory B cell frequencies in these patients (24), although the phenotype of regulatory B cells in this study was tested only for the expression of the key effector molecule IL-10, but not for GrB. In general, there may be both a partial overlap of different regulatory B cell phenotypes, but also different effector types of regulatory B cells, which may be of different relevance in GvHD and other hyperinflammatory conditions. Of course, in the end only clinical studies can give a definite answer to whether or not certain regulatory B cells may have a therapeutic effect in GvHD or autoimmune diseases. Therefore, the GMP-compliant manufacturing of *GraB cells* and other regulatory B cells subsets is considered as an important first step to allow evaluation of their therapeutic potential in autoimmune and other hyperinflammatory diseases within clinical studies. Particularly in light of our observations in HIV patients (16), e*x-vivo* generation and subsequent infusion of autologous or HLA-matched allogeneic regulatory B cells may represent a promising novel therapeutic approach for a variety of these diseases.

Principally, two cell sources are conceivable for *ex-vivo* generation of GrB^+^ regulatory B cells (**Figure 1B**). One major source for donor leukocytes are allogeneic donors, which may be optimally matched to the patient´s leukocytes based on HLA characteristics. Such unrelated HLA-matched donors may be used in case of a preceding hematopoietic stem cell transplantation. Here, the regulatory B cell donor is identical with the original stem cell donor. As a special case, related haploidentical stem cell donors, for example biological children or parents, could also be used as allogeneic regulatory B cell donors. A second major option may be leukocytes harvested from the same patient, who requires treatment with regulatory B cells. The advantage of such autologous regulatory B cells may be a higher specificity as compared with their allogeneic counterparts. For example, in the case of GvHD after stem cell transplantation, alloantigens from the patient can trigger an alloreactive immune response executed by co-transplanted lymphocytes including B cells. In this situation, pre-activated B cells with alloantigenic specificity may be harvested from the patient, re-programmed *ex-vivo* into GrB^+^ regulatory B cells and subsequently be reinfused back into the patient. Main disadvantage of the autologous approach may be disease- and therapy-intrinsic limitations with regard to the number of leukocytes, which can be collected by apheresis.

Phorbol-12-myristate-13-acetate (PMA), also known as 12-O-tetradecanoylphorbol-13-acetate (TPA) is a phorbol ester with a particular analogy to the second messenger diacylglycerol (DAG) (25). Some species of the Daphne family are considered to be natural reservoirs of such phorbol esters (26). Structurally, PMA resembles the tetracyclic ring system of the diterpene alcohol phorbol and is esterified at C12 with the fatty acid myristate. Moreover, PMA is acetylated at C13 (27). These two structural properties are responsible for a strong similarity between DAG and PMA (**Supplementary Figure 1**). Depending on the type of cells and the surrounding milieu, phorbol esters including PMA can have proliferative (28), antiproliferative (29), tumor-promoting (30), antineoplastic (31), inflammatory (32) and antimicrobial (33) effects. A major effect of PMA in B cells is the activation of protein kinase C, which is a central linchpin for gene transcription after B cell activation via its BCR. PMA has previously been used in a multistep stimulation procedure to induce regulatory B10 cells *in vitro* (34–38). In contrast, a connection between *GraB cells* and PMA has so far not been described.

Focus of the present work was to explore PMA as alternative stimulus for IL-21-mediated induction of *GraB cells.* In the originally developed protocol for the generation of *GraB cells*, we used goat-derived xenogeneic F(ab)’2 fragments against the heavy and the light chains of immunoglobulin A, G and M (12). Until now, this stimulus remains an essential part of several protocols described later by other research groups (39, 40). However, for safety reasons, the use of xenogeneic materials in GMP-compliant processes for the manufacturing of human cell products such as *GraB cells* should be avoided as far as possible. Therefore, alternative ways for the activation of the BCR are needed. Due to the structural similarity between PMA and the second messenger DAG we hypothesized that PMA may be able to substitute BCR activation by antibodies or F(ab)’2 fragments, thereby also allowing for an efficient enhancement of IL-21-mediated induction of *GraB cells*.

## Materials and methods

### Human subjects

The present study was approved by the Ethics Committee at Ulm University (registry number 265/15). After acquisition of informed consent, enriched peripheral blood mononuclear cells were obtained from leukapheresis products collected from healthy volunteers using the Spectra Optia^®^ Apheresis System (Terumo BCT, Lakewood, CO, USA). For some experiments, PBMCs were directly isolated from peripheral whole blood.

### Cell isolation and cell culture

Further isolation of peripheral blood mononuclear cells was based on Ficoll density gradient centrifugation. Subsequently, CD19^+^ B cells were positively selected by Magnetic Activated Cell Sorting according to the manufacturer´s instructions (CliniMACS CD19, Miltenyi Biotec, Bergisch Gladbach, Germany). Subsequently, cells were cultured for 48 hours in X-VIVO-15 medium (Lonza Group AG, Basel, Switzerland) on 96-well round bottom plates at a concentration of 1x10^6^/ml and stimulated as indicated. For direct BCR stimulation, affinity purified goat F(ab′)_2_ fragments against human IgA + IgG + IgM (H+L) were used at a final concentration between 3 and 12 µg/ml (Jackson ImmunoResearch Laboratories, West Grove, PA, USA). Human recombinant IL-21 was purchased from Miltenyi Biotec and was used at a final concentration between 25 and 100 ng/ml as indicated. Phorbol-12-myristate-13-acetate (PMA), also known as 12-O-tetradecanoylphorbol-13-acetate (TPA) was purchased from Sigma-Aldrich and was used at a final concentration between 12 and 400 ng/ml as indicated.

### Flow cytometry

Staining of surface markers was performed as described previously. Antibodies to CD20 were purchased from BD Biosciences (San Diego, CA, USA). Granzyme B was detected via intracellular staining. To that purpose, Brefeldin A (BioLegend, San Diego, CA, USA) was added for the last 4 h of incubation at a final concentration of 1 μg/ml. Subsequently, cells were washed with PBS, resuspended in fixation buffer (Cytofix BD Biosciences; San Jose, CA) and incubated for 15 minutes at room temperature. Then, cells were washed with PBS and resuspended in permeabilization buffer (Cytoperm, BD Biosciences; San Jose, CA). PE-labeled antibodies to granzyme B (clone GB11; BD Biosciences; San Jose, CA) or control antibodies were added. Cells were incubated for 15 minutes at 4°C and washed again with PBS. Finally, flow cytometric analyses were performed on a BD FACScan (Becton Dickinson Immunocytometry Systems, San Jose, CA). For data analysis, FlowJo software (Flow Jo Version 10.6., Tree Star, Stanford, CA, USA) was used.

### Statistical analysis

Statistical analysis was performed using Microsoft Excel for Mac, version 16.16.8 and GraphPad Prism, version 9.0.0. Summarized data presented as line graphs are shown as means ± SEM. Data presented as bar graphs are shown as box plots with central horizontal lines representing medians, box edges representing interquartile ranges and whiskers representing the minima and maxima. Dunnett’s multiple comparisons test was performed for comparison of percentages of GrB^+^ regulatory B cells after incubation using different stimuli.

## Results

### Collection of leukocytes for an *ex-vivo* generation of GrB^+^ regulatory B cells

For the present study we used leukocytes from either whole blood or from leukapheresis products collected from healthy donors. Principally, both approaches allow the generation of regulatory B cells with comparable viability and functionality. Nevertheless, due to the higher number of cells required for therapy, future development of a GMP-compliant regulatory B cell manufacturing process will most likely be based on leukapheresis products as starting material. In order to quantify the number of GrB^+^ regulatory B cells, which may be obtained from an average leukapheresis product, we collected leukocytes from 20 healthy donors and isolated CD19^+^ B cells using a GMP-compliant magnetic isolation kit. Leukocyte and B cell counts as well as further isolation parameters for all 20 donors are listed in **Supplementary Table 1**. The average number of B cells, which may be obtained from a full 250 ml leukapheresis product, was 167.1 x 10^6^ cells, at an average viability of 95.1% and an average purity of 98.4%. These data demonstrate that isolation and generation of GrB^+^ regulatory B cells from healthy donors using leukapheresis is both feasible and provides sufficiently high B cell numbers for further manufacturing of regulatory B cells.

### *Ex vivo* generation of GrB^+^ regulatory B cells by phorbol-12-myristate-13-acetate

**Figure 2:**
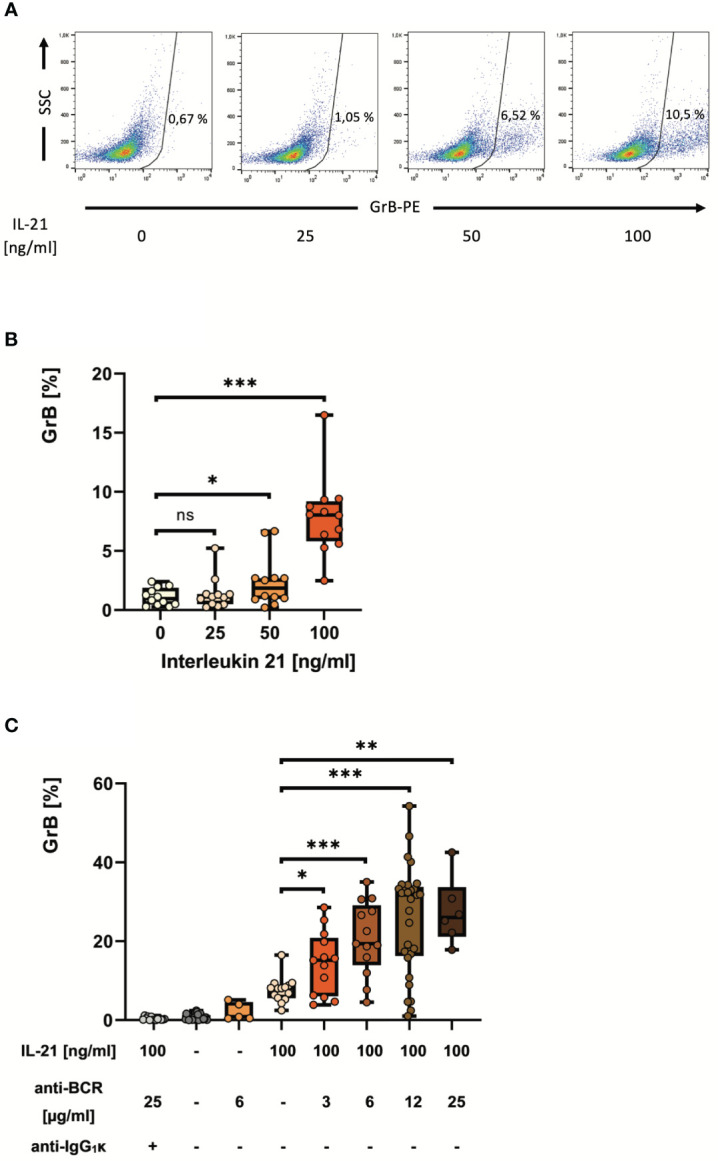
IL-21 induces and antibodies to the B cell receptor strongly enhance differentiation of pre-activated B cells into GrB^+^ *GraB cells.* B cells from healthy donors were isolated from PBMC using magnetic beads directed against CD19. Subsequently, B cells were incubated for 48 hours in the presence of increasing concentrations of IL-21 and anti-BCR as indicated. Then, B cells were harvested, stained for intracellular GrB and analyzed by flow cytometry. **(A)** Dot plots show percentages of GrB^+^ GraB cells from one representative experiment out of n > 10. **(B, C)** Box plots show average percentages of GrB^+^ *GraB cells* from n > 10 individual experiments as indicated, each dot indicates a result from one individual experiment. Box central horizontal lines indicate medians, box borders represent IQR, whiskers indicate minima and maxima. Significance levels were *** p < 0.0005, ** p < 0.005 and * p < 0.05. Anti-BCR, activating antibodies against the constant part of the B Cell Receptor; GrB, Granzyme B; IgG, Immunoglobulin G; IL-21, Interleukin 21; IQR, interquartile ranges; ns, not significant; PBMC, Peripheral Blood Mononuclear Cells; PE, Phycoerythrin; SSC, Side Scatter.

As outlined above, three signals determine whether B cells differentiate into plasma cells or *GraB cells* (**Figure 1A**). For *GraB cell* development, the IL-2 family cytokine IL-21 plays a central role by triggering GrB induction in pre-activated B cells in a dose-dependent manner (**Figures 2A, B**). Nevertheless, only in the presence of additional activation of the BCR, a significant number of pre-activated B cells change its phenotype into GrB^+^ regulatory B cells (**Figure 2C**). Since for safety reasons the use of xenogeneic polyclonal antibodies or F(ab′)_2_ fragments directed against the BCR is not possible in a GMP-compliant process, alternative activation of the pathways downstream of the BCR are needed.

**Figure 3:**
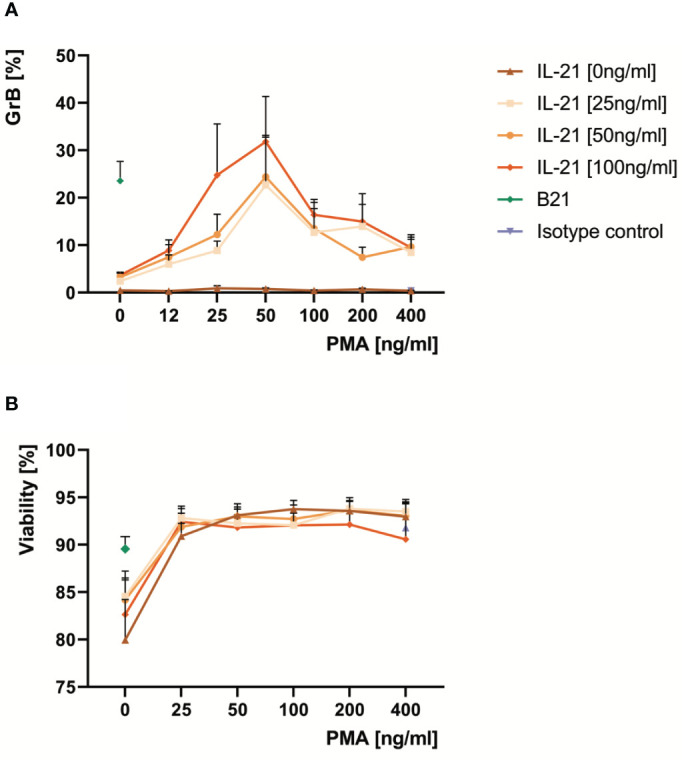
Optimal doses of PMA differentiate pre-activated B cells into GrB^+^ *GraB cells* and improve their viability *ex vivo.* B cells from at least ten healthy donors were isolated from PBMC using magnetic beads directed against CD19. Subsequently, B cells were incubated for 48 hours in the presence of increasing concentrations of IL-21 and PMA as indicated. Anti-BCR at 12 µg/ml and IL-21 at 100 ng/ml served as positive control. Then, B cells were harvested, stained for intracellular GrB and analyzed by flow cytometry. **(A)** Line graphs show mean percentages of GrB^+^ *GraB cells* from n > 10 individual experiments as indicated. **(B)** Line graphs show mean viability of GraB cells at the end of the cell culture period based on morphological criteria. Error bars indicate SEM. Anti-BCR, activating antibodies against the constant part of the B Cell Receptor; B21, Anti-BCR + IL-21; GrB, Granzyme B; IL-21, Interleukin 21; PMA, Phorbol Myristate Acetate; SEM, Standard Error of the Mean.

**Figure 4:**
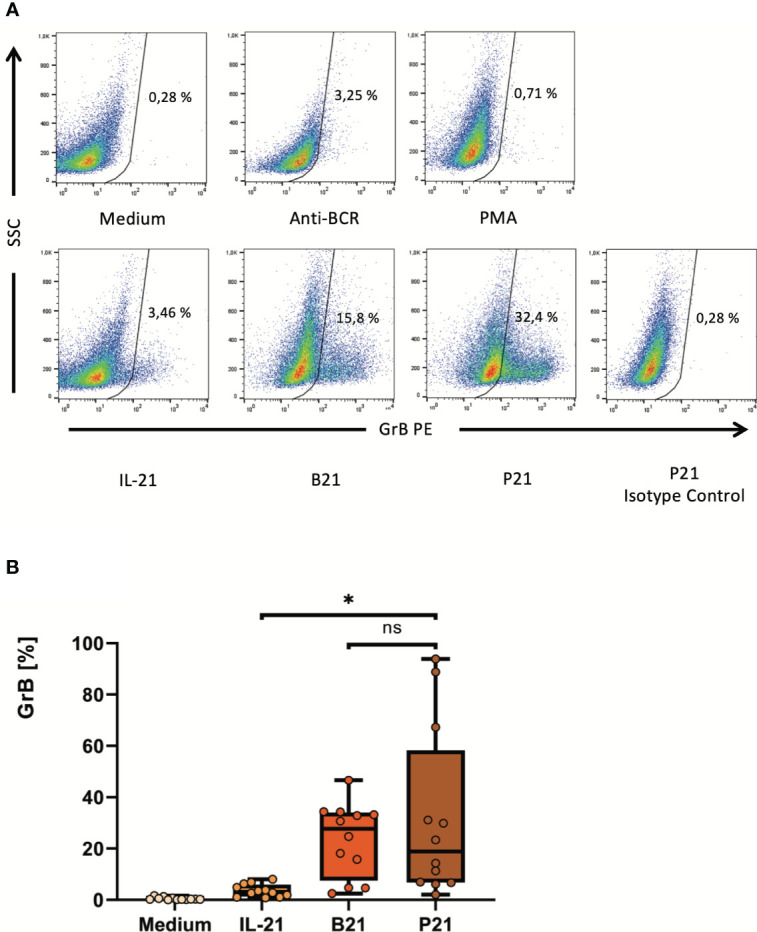
PMA may replace activating antibodies against the B cell receptor to enhance IL-21-dependent *ex-vivo* differentiation of pre-activated B cells into GrB^+^ *GraB cells.* B cells were isolated from PBMC using magnetic beads directed against CD19. Subsequently, B cells were incubated for 48 hours in the presence of different combinations of IL-21 at 100 ng/ml, anti-BCR at 12 µg/ml and PMA at 50 ng/ml as indicated. Then, B cells were harvested, stained for intracellular GrB and analyzed by flow cytometry. **(A)** Dot plots show percentages of GrB^+^ GraB cells from one representative experiment out of n > 10. **(B)** Box plots show average percentages of GrB^+^ *GraB cells* from n > 10 individual experiments as indicated. Box central horizontal lines indicate medians, box borders represent IQR, whiskers indicate minima and maxima. Significance level was * p < 0.05. Anti-BCR, activating antibodies against the constant part of the B Cell Receptor; B21, Anti-BCR + IL-21; GrB, Granzyme B; IL-21, Interleukin 21; IQR, interquartile ranges; ns, not significant; P21, PMA + IL-21; PBMC, Peripheral Blood Mononuclear Cells; PE, Phycoerythrin; PMA, Phorbol Myristate Acetate; SSC, Side Scatter.

In order to find alternatives for a direct activation of the BCR, a deeper look into the signaling cascades downstream of the BCR is required (**Supplementary Figure 2**). One key downstream response after binding of antigen to a specific B cell receptor on the B cell surface is the activation of protein kinase C (PKC) by the second messenger diacylglycerol (DAG). Importantly, the effect of DAG can be bypassed by the alternative PKC activator phorbol myristate acetate (PMA). Therefore, we hypothesized that the synergistic effect of BCR activation on IL-21- induced GrB induction in B cells may be substituted by PMA. We performed a series of experiments with isolated B cells, which were incubated for 48 hours at increasing concentrations of IL-21 and PMA. As expected we found that PMA and IL-21 were able to induce substantial amounts of GrB in B cells, with an optimal PMA concentration identified at 50 ng/ml (**Figure 3A**). As for antibodies against the BCR (anti-BCR), we found that the presence of PMA resulted in a stabilization of B cell viability during ex vivo cell culture, which ranged between 90 and 95% (**Figure 3B**). When directly comparing optimal concentrations of anti-BCR and IL-21 with optimal concentrations of PMA and IL-21, we found that both were similarly effective in inducing GrB^+^ B cells (**Figures 4A, B**).

## Discussion

Here we demonstrated that phorbol-12-myristate-13-acetate (PMA), also known as 12-O-tetradecanoylphorbol-13-acetate (TPA), is a potent enhancer of IL-21-induced differentiation of pre-activated B cells into GrB^+^ regulatory B cells (*GraB cells*). The percentage of GrB^+^ B cells as well as absolute expression levels of GrB after stimulation of pre-activated B cells with IL-21 and PMA was not significantly lower compared to stimulation with IL-21 and goat-anti-human F(ab)’2 fragments against the constant part of the B cell receptor (anti-BCR). Maximum induction was achieved at concentrations of 50 ng/ml PMA and 100 ng/ml IL-21. On average, more than one third of B cells expressed high levels of GrB after 48 hours stimulation, suggesting that under steady-state conditions around 30% of B cells are in a pre-activated state in healthy individuals. This appears plausible since previously we could show that during acute infections, for example in HIV patients, up to 100% of B cells can be in a pre-activated state and may thus be induced to express GrB *ex-vivo* (13, 16). Of note, B cell viability remained between 90 and 95% within the tested concentration range of PMA up to 400 ng/ml. As previously shown for anti-BCR, also for PMA the addition of IL-21 was elementary for the generation of *GraB cells*. In contrast, CD40 ligand suppressed their induction. Therefore, these findings support once more our hypothesis that *in-vivo* the generation of *GraB cells* may occur in the presence of incompletely activated T cells (13, 15, 16, 20).

As hypothesized above, the effect of PMA on the generation of *GraB cells* may be due to its structural analogy with diacylglycerol (DAG). As a second messenger, DAG activates protein kinase C (PKC) in B cells and subsequently regulates essential transcription factors such as NFAT, Myc, NF-κB and AP-1 (41). These transcription factors in turn affect gene expression including that of the gene for GRB. It is not surprising that as a consequence PMA can also initiate PKC activation with subsequent GrB degranulation in regulatory T cells and in NK cells, where transcription of the GRB gene is subject to PKC-regulated NF-κB (42). Therefore, from a functional point of view, induction of *GraB cells* by PMA, as observed in the present study, appears plausible.

PMA is not an entirely unknown player in the field of regulatory cells. Previously, PMA was also tested for the generation of B10 cells (34–38). According to Tedder and colleagues, CD40 ligation or TLR stimulation can induce pro-B10 cells in a first maturation step. Although the impact of the BCR in this process has not been conclusively clarified yet, the formation of competent IL-10-secreting B10 cells is promoted *in vitro* by stimulation with either PMA, the TLR4 agonist LPS, or ionomycin (34–38). Therefore, for the generation of murine B10 cells, a two-stage stimulation procedure was developed consisting of 48 hours stimulation with CD40 ligand, followed by 5 hours stimulation with PMA, LPS and ionomycin. This regimen results in the induction of murine regulatory B10 cells with a CD1d^+^CD5^+^CD19^hi^ phenotype.

As for the induction of *murine* regulatory B10 cells, a multiphase model was also discussed for the development of *human* B10 cells. Daien and colleagues found that patients with rheumatoid arthritis showed a quantitative deficit of B10 cells compared to healthy controls (43). They were able to induce B10 cells *ex-vivo* by first stimulating peripheral B cells with CpG, anti-BCR and CD40L for 24 hours, followed by a second stimulation phase with PMA, ionomycin and brefeldin A for 4 hours. Besides, PMA was used in re-stimulation experiments to maintain IL-10 production in human B10 cells. In these experiments, a small fraction of formerly IL10-negative B cells could be stimulated to produce IL-10 (44). The studies described above suggest that PMA may have a comprehensive effect on B cells, which results in the induction of B cells with various regulatory functions. This and the obvious functional similarities between B10 and *GraB cells* imply a transferability of the trigger PMA for the induction of a GrB^+^ phenotype in B cells. Regardless of the exact phenotype of regulatory B cells induced by PMA and possible overlaps in regulatory B cell phenotypes, in the end only clinical studies with GMP-compliantly manufactured regulatory B cells will be able to show whether or not such cells may have a therapeutic effect on hyperinflammatory conditions including GvHD or autoimmune diseases.

The potential clinical use of PMA to generate regulatory B cells must be carefully evaluated due to its pleiotropic mode of action. Although PMA acts as a driving force to induce the differentiation of B cells to regulatory *GraB cells*, it can also cause the release of a variety of cytokines by further immune cells including CD4^+^ T cells. Among others, PMA induces IL-21, thereby triggering the same cytokine that is indispensable for the induction of *GraB cells* (17, 45). Based on the effects described and a longer half-life of PMA compared with DAG, a pronounced and extended potency of PMA to induce *GraB cells* could be expected if applied systemically. On the other hand, the release of further cytokines including IL-4, IL-6, IL-8, TNF-α, and IFN-γ upon *in-vivo* application of PMA must be considered as well (46–48). The consequences were observed in a clinical trial in 1998 (49, 50). In this trial, 12 patients with refractory malignant disease (CML, AML, MDS) were treated with PMA/TPA alone or in combination with cytosine arabinoside and vitamin D3. PMA was applied at 0.25 - 1 mg per week as an infusion in 200 ml saline. Subsequently, mild flu-like symptoms were observed, which included chills, fevers and sweats for 3 - 5 hours. In addition, dyspnea, hemoglobinuria, proteinuria and bleeding signs were observed (49, 50). Two follow-up trials reported similar adverse effects (51, 52). Since more severe consequences such as circulatory shock, consumption coagulopathy and multiorgan failure may not be excluded in the setting of a cytokine release syndrome, direct systemic use of PMA may be considered only after thorough risk-benefit evaluation.

Another possible risk associated with PMA is its mitogenic potential. Phorbol esters like PMA are classified as tumor-promoting substances (30). Its carcinogenic potential was demonstrated in a multistage murine skin tumor formation model. The polycyclic aromatic hydrocarbon 7,12-dimethylbenzoanthracene (DMBA) served as a sub-carcinogenic stimulus, thereby causing initial cell transformation. Recurrent application of PMA as a tumor-promoting growth stimulus subsequently led to the formation of papillomas (30, 53). Malignant progression occurred only when additional sub-carcinogenic doses of DMBA were added. On the other hand, in some malignancies PMA also showed tumor-suppressive effects. For example, in a study with prostate cancer cells, PMA was documented to upregulate pro-apoptotic (JNK, p53) and to inhibit proliferation-promoting signals (E2F1) (31). Moreover, antileukemic effects were demonstrated in the context of myeloid leukemias both *in vivo* and *in vitro* (54, 55). After translation of these results into the clinic, intravenous application of PMA/TPA in patients with CML, AML or MDS indeed caused a reduction of leukemic blasts. In some cases, this resulted in a partial remission and an improvement in the patients’ quality of life (49–52). Similar anti-tumor effects of PMA have been described for certain B-cell malignancies *in vitro*. For example, PMA and ionomycin induced apoptosis in OCI-LY1 cells of diffuse large B-cell lymphoma (DLBCL) (56).

The originally developed protocol for the generation of *GraB cells* used goat-derived xenogeneic F(ab)’2 fragments against the BCR (12), which until today remain an essential part of respective manufacturing protocols (39, 40). On the other hand, the use of xenogeneic material for the manufacturing of advanced therapy medicinal products (ATMPs) should be avoided as far as possible to prevent adverse immune reactions in the patient as well as potential transmission of so far unknown diseases. Therefore, after careful consideration of the risks and benefits, the use of PMA/TPA appears to be a reasonable alternative for *ex-vivo* manufacturing of *GraB cells*.

In summary, *ex-vivo* manufacturing of autologous or allogeneic granzyme B-expressing B cells (*GraB cells*) based on an autologous or allogeneic (e.g. haploidentical or 10/10-HLA-matched) leukapheresis appears feasible. B cells from these apheresis products may be enriched in a clean room environment using Good Manufacturing Practice (GMP)-compliant kits containing antibodies against human CD3 and CD19. Enriched B cells may then be stimulated for 48 hours in an incubator (37°C, 5% CO_2_, > 95% humidity) in the presence of 50 ng/ml PMA/TPA and 100 ng/ml recombinant human IL-21. During this period, pre-activated B cells can differentiate into *GraB cells* and acquire their regulatory properties. Then, the resulting *GraB cells* may be harvested and washed several times to remove remaining PMA. Before release as an ATMP, additional quality controls including proof of absence of relevant PMA concentrations in the product are required according to the GMP guidelines of the European Commission to ensure product quality and safety. These include microbiological testing including mycoplasma and endotoxin testing as well as PMA testing. Finally, the *GraB cell*-containing cell product can be (re-)transfused into the patient. Particularly the establishment of a reliable method to detect remaining PMA in the final medicinal product will be one the last hurdles before a GMP-compliant manufacturing process can be implemented.

## Data availability statement

The raw data supporting the conclusions of this article will be made available by the authors, without undue reservation.

## Author contributions

BJ designed the study. BJ and JV performed literature research. JV, CM, AF and CL performed the experiments. BJ conceptualized and supervised the analytics. JV, CM, AF and LG collected data, JV and BJ analyzed and interpreted data and prepared figures. BJ, PR, HS and DF provided key research tools. BJ and JV wrote the manuscript. BJ, JV and CL verified the underlying data. All authors contributed to the article and approved the submitted version.
