# Supplementary material for: Phorbol-12-myristate-13-acetate is a potent enhancer of B cells with a granzyme B+ regulatory phenotype
Source: Front Immunol. 2023 Jul 27;14:1194880. doi: 10.3389/fimmu.2023.1194880 (PMC10426744; doi:10.3389/fimmu.2023.1194880)
Supplement: Supplementary file 1 [file DataSheet_1.pdf]

Experiment	Leukocyte concentration in apheresis product [*10³/µl]	Number of PBMCs isolated [*10⁶]	Number of BCs isolated [*10⁶]	Estimated number of BCs in full 250 ml apheresis product [*10⁶]	Expected percentage BCs / PBMCs as estimated by FACS analysis [%]	Effective percentage BCs / PBMCs isolated [%]	BC viability [%]	BC purity [%]
1	26.4	800	23.8	141.67	7.29	2.98	96.10	98.30
2	19.8	680	21.6	120.00	5.16	3.18	88.00	98.80
3	73.1	1500	29.1	291.25	13.10	1.94	98.90	99.70
4	54.4	1500	36.0	100.00	17.60	2.40	95.90	98.40
5	23.2	875	16.7	83.60	7.76	1.91	93.60	99.30
6	56.7	653	13.8	229.17	12.60	2.11	97.00	97.10
7	23.9	820	17.9	112.00	6.90	2.19	92.50	97.00
8	19.6	910	5.2	21.67	1.97	0.57	95.70	98.80
9	43.2	1024	31.8	176.56	7.40	3.10	94.80	99.40
10	44.4	591	10.2	150.00	7.81	1.73	93.60	98.60
11	55.4	520	12.9	107.50	6.85	2.48	95.20	99.10
12	22.4	600	17.3	143.75	5.95	2.88	96.40	99.40
13	14.5	304	3.3	34.38	3.23	1.09	95.70	97.40
14	37.5	473	16.0	266.00	9.13	3.37	94.90	99.10
15	20.3	912	44.4	184.88	9.96	4.87	96.10	98.40
16	19.6	769	26.3	146.11	9.26	3.42	98.60	98.00
17	20.0	615	14.4	79.86	9.95	2.34	89.50	98.50
18	40.8	1000	96.0	480.00	11.90	9.60	96.20	98.20
19	49.7	800	47.4	237.00	9.61	5.93	96.40	96.70
20	49.7	800	47.4	237.00	9.61	5.93	96.40	96.70
**Mean**	**35.74**	**807.30**	**26.57**	**167.12**	**8.65**	**3.20**	**95.08**	**98.35**
**SEM**	**3.87**	**68.34**	**4.79**	**23.87**	**0.81**	**0.47**	**0.61**	**0.21**

Supplementary Table 1. Performance parameters for B cell isolation from leukapheresis products

Mononuclear cells from 20 healthy donors were collected using the Spectra Optia® Apheresis System in a total volume of 250 ml each. A fraction of this volume between 20 and 90 ml was further processed as described in Materials and Methods, resulting in a 98% pure B cell population with an estimated average total number of 167 x 10^6 B cells per leukapheresis product at an average viability of more than 95%. Table 1 shows individual performance parameters for PBMC and BC isolation including expected and effective percentages of BCs isolated from total PBMCs as well as BC viability and BC purity. Abbreviations: BC = B cell, FACS = Fluorescence-Activated Cell Sorting (flow cytometry), PBMC = peripheral blood mononuclear cells.
