# Supplementary material for: Phorbol-12-myristate-13-acetate is a potent enhancer of B cells with a granzyme B+ regulatory phenotype
Source: Front Immunol. 2023 Jul 27;14:1194880. doi: 10.3389/fimmu.2023.1194880 (PMC10426744; doi:10.3389/fimmu.2023.1194880)
Supplement: Supplementary file 2 [file DataSheet_2.pdf]

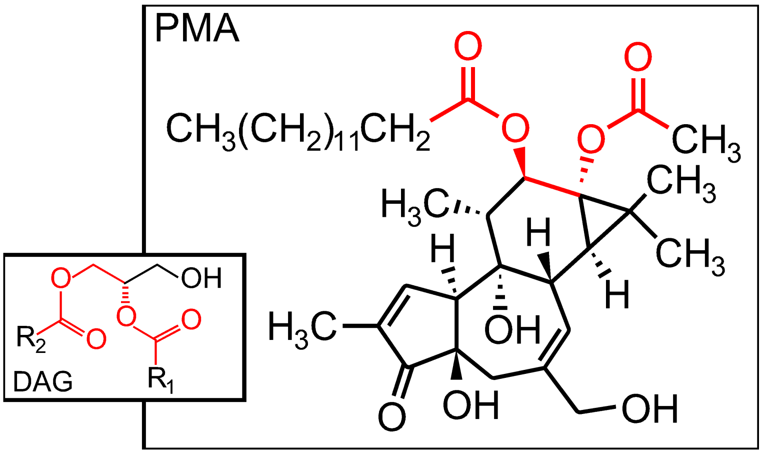


**Supplementary Figure 1. Analogy between diacylglycerol and phorbol-12-myristate-13-acetate**

The chemical structures of PMA (right) and DAG (left) are compared. The main structural similarity, the diacyl residue, is highlighted in red. R1 and R2 denote possible variable fatty acid residues of DAG such as palmitoyl, arachidonoyl or stearoyl. The illustration of the chemical compounds was created using AutoCAD 2016, Version 20.1. Abbreviations: DAG = Diacylglycerol, PMA = Phorbol Myristate Acetate.

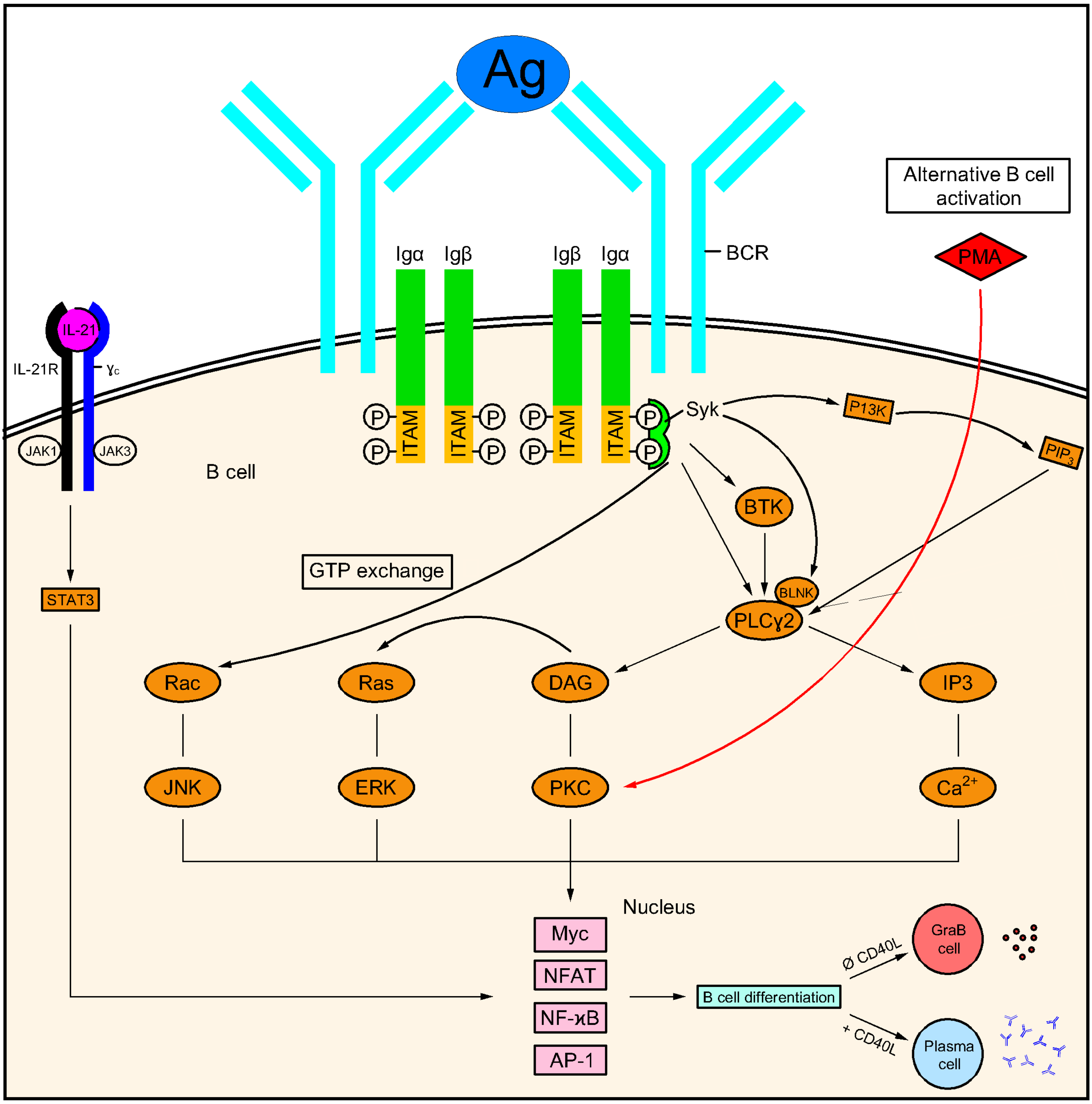


**Supplementary Figure 2. Alternative induction of GrB⁺ *GraB cells* by PMA based on its structural analogy to the downstream second messenger DAG**

The cartoon schematically outlines the signaling cascades, which are set in motion after binding of an antigen to a specific B cell receptor. In this process a key downstream response after binding of antigen is the activation of PKC by the second messenger DAG. PMA, highlighted in red, is a potential alternative activator of PKC, thereby bypassing the effect of DAG. Abbreviations: Ag = Antigen, BCR = B Cell Receptor, CD40L = Cluster of Differentiation 40 Ligand, DAG = Diacylglycerol, IL-21 = Interleukin 21, IL-21R = IL-21 Receptor, JAK = Janus Kinase, PKC = Protein Kinase C, PMA = Phorbol Myristate Acetate.
